# The changes in cardiovascular disease risk factors after the implementation of the package of essential non-communicable disease control

**DOI:** 10.34172/jcvtr.33127

**Published:** 2024-09-20

**Authors:** Ali Reza Pouramini, Fatemeh Kafi, Reza Khadivi

**Affiliations:** ^1^Community and Family Medicine Department, Medical Faculty, Isfahan University of Medical Sciences, Isfahan, Iran; ^2^Medical Mycology and Bacteriology Research Center, Kerman University of Medical Sciences, Kerman, Iran

**Keywords:** Cardiovascular diseases, Diabetes mellitus, Hypercholesterolemia, Hyperlipidemias, Hypertension, Obesity

## Abstract

**Introduction:**

The Package of Essential Non-Communicable (PEN) Disease Control was implemented in the primary healthcare system to manage cardiovascular disease (CVD) risk factors in Iran. This study aimed to evaluate the status of CVD risk factor control following the implementation of the PEN.

**Methods:**

This historical cohort study was conducted among 60-65-year-old residents to compare hypertension (HTN) control via mean systolic blood pressure (SBP) and diastolic blood pressure (DBP), diabetes mellitus (DM) control via fasting blood sugar (FBS) and HbA1C tests, hyperlipidemia control via serum cholesterol and triglyceride levels, and overweight and obesity via body mass index (BMI) measurement in 2016 (before the implementation of the PEN project) and 2021 (after 5 years).

**Results:**

A total of 1,583 residents with a mean age of 62.32±1.70 years were included in the study. In 2021, compared to 2016, there was a significant decrease in the relative frequency of residents with high SBP from 13.7% to 9.3%, high DBP from 11.3% to 3.4%, FBS≥126 mg/dL from 25.6% to 19.7%, and BMI≥30 from 25.7% to 23.4%. Additionally, the mean DBP and the mean serum levels of FBS among all participants decreased significantly. However, the relative frequency of residents with total cholesterol≥200 mg/dL increased significantly from 11.5% to 14.2%.

**Conclusion:**

Following the implementation of the PEN, the control of HTN, DM, and obesity improved among 60-65-year-old residents. However, the control of hypercholesterolemia did not improve.

## Introduction

 Cardiovascular diseases (CVDs) are currently the leading causes of mortality, morbidity, and disability.^1^ CVDs account for 46% of all deaths and at least 30% of the disease burden in Iran.^2^ Hypertension (HTN), diabetes mellitus (DM), hypercholesterolemia, hypertriglyceridemia, smoking, obesity, and physical inactivity are the primary risk factors for CVD.^3,4^ It is estimated that more than 75% of premature cardiovascular events are preventable. Consequently, the World Health Organization (WHO) proposed a global action plan to reduce premature deaths due to non-communicable diseases (NCDs) by 25% by 2025, with a focus on CVDs. The WHO developed the Package of Essential Non-Communicable Diseases (PEN) to prevent and control four main NCDs: CVDs, cancers, and chronic respiratory diseases.^5^ Iran’s Package of Essential Non-Communicable Diseases (Ira-PEN) was designed by the Iranian Ministry of Health and Medical Education to achieve the long-term WHO goals of preventing and reducing the risk of four main NCDs among the 30 to 70-year-old population, starting in 2016. Ira-PEN was implemented as a pilot program in the counties of Shahreza, Naghadeh, Baft, and Maragheh.^6^

 According to the PEN guidelines, health workers actively engaged residents over 30 years old, taking their medical histories related to cardiovascular disease, DM, HTN, smoking, alcohol consumption, and family history of premature death due to CVD. They assessed weight, height, abdominal circumference, and systolic and diastolic blood pressure, and measured serum fasting blood sugar (FBS), total cholesterol, low-density lipoprotein (LDL), high-density lipoprotein (HDL), and triglycerides (TG) in a designated partner laboratory regularly. Individuals with elevated blood pressure in two consecutive measurements or abnormal serum FBS, TG, HDL, or LDL in the initial evaluation were referred to a general physician for further examination and workup. During each follow-up visit, after assessing CVD risk factors, a cardiovascular event risk assessment card was completed according to the WHO/ISH risk chart, classifying individuals into four risk groups: low (risk less than 10%), medium (10%-19%), high (20%-29%), and very high (> 30%). Follow-up schedules were conducted annually for the low-risk group, every 9 months for the medium-risk group, every 6 months for the high-risk group, and every 3 months for the very high-risk group. Follow-up schedules were revised based on the updated risk level at the most recent visit. During follow-up visits, appropriate counseling and training were provided to enhance medical adherence and promote healthy behaviors, including following a healthy diet, engaging in sufficient physical activity, and quitting smoking and alcohol consumption according to goal-setting strategies aimed at encouraging behavior change.^6^

 This study aims to compare the control of CVD risk factors 5 years after the implementation of the PEN in 2021 with the baseline data from 2016 (before the PEN project implementation).

## Materials and Methods

###  Study Design & Data Sources

 This historical cohort study aims to compare data related to CVD risk factors, including body mass index (BMI), blood pressure (systolic and diastolic), and serum values of FBS, HbA1C, HDL, LDL, and TG in 60-65-year-old citizens in Shahreza, Isfahan, Iran, between 2016 and 2021. Although the PEN plan was designed for the population over 30 years old, priority was given to 60-65-year-old citizens during the first year of Ira-PEN implementation. Data collected from this population in 2016 were accurately recorded in the integrated electronic health file system (SIB software system in Persian) and used as baseline data for our study. Data were collected using a census sampling method.

###  Inclusion and Exclusion Criteria

 Clinical data from 60-65-year-old inhabitants of the Shahreza district of both sexes, with no history of CVD (acute myocardial infarction, stroke, heart failure, or chronic heart disease), as well as laboratory data from performed biochemical tests, were included. Health files of Shahreza district inhabitants with a CVD history, those out of the specified age range, files with missing information (> 20%), or those not residing in Shahreza and registered as guests in the health system were excluded from the study.

###  Data Collection and Measurements

 According to PEN guidelines, health staff in health centers assessed weight, height, abdominal circumference, and systolic and diastolic blood pressure through physical examination, and measured serum FBS, HbA1C, LDL, HDL, TG, and total cholesterol in an exclusive partner laboratory regularly. The data were subsequently recorded in the electronic health file (SIB software system).

 Health staff used calibrated scales and stadiometers to assess weight and height with an error margin of ± 0.1 kilograms and ± 0.1 centimeters, respectively. All inhabitants were measured in light clothing and without shoes. BMI was calculated using the formula weight / (height)^2^ kg/m^2^, with obesity defined as BMI ≥ 30 kg/m^2^.

 A calibrated mercury sphygmomanometer (Richter Aneroid, Germany) with appropriate cuff sizes was used twice, at a 5-minute interval, to assess blood pressure. Participants were asked to rest for at least 15 minutes, and blood pressure was taken in a sitting position. For individuals with normal blood pressure (less than 120/80 mm Hg), blood pressure was measured at least once every two years. Those with systolic or diastolic blood pressure higher than 140 mm Hg and 90 mm Hg, respectively, were referred to a general physician for consultation and further examination.

 According to PEN guidelines, diabetes mellitus was defined as FBS ≥ 126 mg/dL, HbA1C ≥ 6.5%, or random plasma glucose ≥ 200 mg/dL. The blood lipid profile included measurements of total cholesterol, HDL cholesterol, LDL cholesterol, and triglycerides, measured in milligrams per deciliter (mg/dL). Hypertriglyceridemia was defined as triglycerides ≥ 150 mg/dL, and hypercholesterolemia was defined as total cholesterol ≥ 200 mg/dL. The SIB dataset provided the appropriate schedule for the next follow-up according to the guidelines outlined in the Ira-PEN project.

###  Statistical analysis

 All data from this study were analyzed using IBM SPSS version 26. Descriptive data are reported as mean ± standard deviation (SD), frequency, and percentage (%). The McNemar test and Paired Samples T-test were used for the analysis of qualitative and quantitative values, respectively. A p-value lower than 0.05 was considered significant.^6^

## Results

 Of the total 6,896 inhabitants aged 60-65 years, 1,583 individuals (22.96%) with a mean age of 62.32 ± 1.70 years were included in this study in 2016. Among the participants, 397 (25.1%) lived in rural areas, and 1,186 (74.9%) lived in urban areas. The percentage of men decreased from 47.1% in 2016 to 40.8% in 2021, while the percentage of women increased from 52.9% to 59.2%. The McNemar test indicated that the gender distribution difference was significant in both 2016 and 2021 (*P* value < 0.001) (Table 1).

**Table 1:** Gender distribution and the mean cardiovascular disease risk factors of participants in 2016 and 2021

**Variable**	**Sex**	**2016**	**2021**	***P*** **-value**
Gender	Male	746 (47.1%)	618 (40.8%)	< 0.001*
	Female	837 (52.9%)	895 (59.2%)	
	Total	1583 (100%)	1513 (100%)	
BMI (kg/m2)	Male	25.51 ± 4.13	25.45 ± 4.12	0.735^**^
	Female	28.22 ± 5.06	28.05 ± 4.89	0.344^**^
	Total	27.03 ± 4.80	26.93 ± 4.78	0.494^**^
FBS (mg/dL)	Male	120.82 ± 49.30	110.49 ± 38.68	0.058^**^
	Female	119.58 ± 60.18	112.37 ± 40.91	0.082^**^
	Total	119.93 ± 57.28	111.85 ± 40.26	0.011^**^
HbA1C (%)	Male	7.83 ± 2.11	7.35 ± 2.07	0.160^**^
	Female	7.17 ± 1.67	7.16 ± 1.72	0.954^**^
	Total	7.37 ± 1.83	7.21 ± 1.82	0.369^**^
SBP (mm Hg)	Male	117.88 ± 16.45	117.34 ± 13.46	0.480^**^
	Female	116.29 ± 16.35	117.85 ± 13.96	0.018^**^
	Total	116.99 ± 16.41	117.65 ± 13.76	0.186^**^
DBP (mm Hg)	Male	74.68 ± 10.62	73.18 ± 8.67	0.003^**^
	Female	72.61 ± 10.8	72.28 ± 9.20	0.449^**^
	Total	73.46 ± 10.77	72.63 ± 9.01	0.007^**^
Total cholesterol (mg/dL)	Male	148.4 ± 33.22	156.0 ± 34.85	< 0.001^**^
	Female	160.9 ± 39.14	166.9 ± 39.04	< 0.001^**^
	Total	155.5 ± 37.20	162.6 ± 37.82	< 0.001^**^
LDL (mg/dL)	Male	102.38 ± 32.87	99.59 ± 43.37	0.785^**^
	Female	114.00 ± 32.84	111.71 ± 32.38	0.602^**^
	Total	111.34 ± 33.14	109.02 ± 35.24	0.560^**^
HDL (mg/dL)	Male	41.06 ± 9.71	42.16 ± 8.07	0.620^**^
	Female	45.35 ± 10.12	44.69 ± 11.22	0.622^**^
	Total	44.32 ± 10.18	44.15 ± 10.64	0.878^**^
TG (mg/dL)	Male	151.87 ± 81.37	182.18 ± 122.04	0.122^**^
	Female	160.36 ± 74.23	159.87 ± 89.86	0.955^**^
	Total	158.30 ± 75.89	164.56 ± 97.54	0.462^**^

* Using McNemar test ** Using Paired Samples T-test BMI: Body Mass Index, FBS: Fasting Blood Sugar, SBP: Systolic Blood Pressure, DBP: Diastolic Blood Pressure, LDL: Low-density Lipoprotein, HDL: High-density Lipoprotein, TG: Triglycerides.

 Using the McNemar test, it was demonstrated that from 2016 to 2021, the relative frequency of citizens with high SBP significantly decreased from 13.7% to 9.3% (*P *value < 0.001). Similarly, the relative frequency of citizens with high DBP significantly decreased from 11.3% to 3.4% (*P* value = 0.008). The relative frequency of participants with FBS ≥ 126 mg/dL significantly decreased from 25.6% to 19.7% (*P* value < 0.001). The relative frequency of citizens with HbA1C ≥ 6.5% significantly decreased from 70% to 60.87% (*P* value = 0.002), and the relative frequency of citizens with BMI ≥ 30 significantly dropped from 25.7% to 23.4% (*P* value < 0.001).

**Figure 1:**
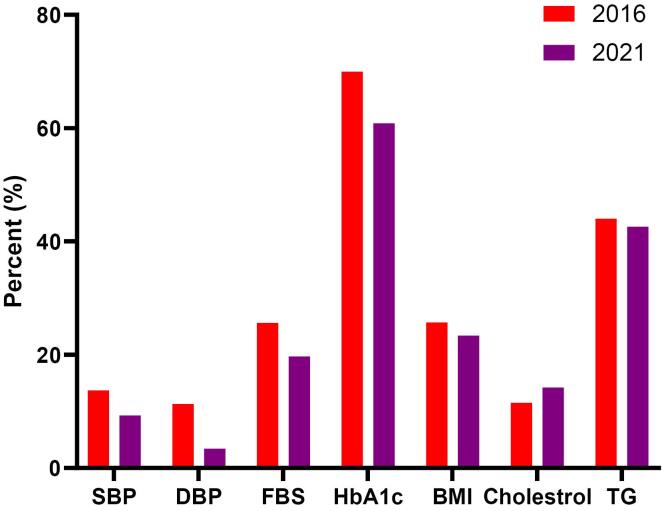
Changes in the relative frequency of citizens with cardiovascular disease risk factors after the implementation of the PEN; SBP: Systolic Blood Pressure ≥ 140 mmHg, DBP: Diastolic Blood Pressure ≥ 90 mmHg, FBS: Fasting Blood Sugar ≥ 126 mg/dl, Hb1Ac ≥ 6.5%, BMI: Body Mass Index ≥ 32 kg/m2, Total Cholesterol ≥ 200 mg/dl, TG: Triglycerides ≥ 150 mg/dl

 Conversely, the relative frequency of citizens with total cholesterol ≥ 200 mg/dL significantly increased from 11.5% to 14.2% (*P* value < 0.001), but the relative frequency of citizens with triglycerides ≥ 150 mg/dL decreased insignificantly from 44.0% to 42.6% (*P* value = 0.228) (Figure 1).

 On the other hand, using the Paired-Samples T-test revealed that the mean serum level of FBS decreased significantly from 119.93 ± 57.28 to 111.85 ± 40.26 mg/dL (*P* value = 0.011), and the mean diastolic blood pressure significantly decreased from 73.46 ± 10.77 to 72.63 ± 9.01 mmHg (*P* value = 0.007). However, the mean serum level of total cholesterol significantly increased from 155.6 ± 37.13 to 162.6 ± 37.82 mg/dL (*P* value = 0.001).

 The increase in mean SBP was insignificant overall (from 116.99 ± 16.41 to 117.65 ± 13.76 mmHg). Nonetheless, the mean SBP in women increased significantly from 116.29 ± 16.35 to 117.85 ± 13.96 mmHg (*P* value = 0.018) (Table 1).

## Discussion

 This study aimed to evaluate the status of CVD risk factor control after implementing the PEN in a pilot county. Our findings indicate significant reductions in the relative frequency of obesity, high SBP, high DBP, FBS ≥ 126 mg/dL, and HbA1C ≥ 6.5%, as well as significant decreases in the mean serum levels of FBS and mean DBP among 60-65-year-old citizens. These results align with the WHO’s goal of a 25% reduction in NCD risk factors by 2025.^5^ Additionally, our findings are consistent with outcomes from PEN interventions in Bhutan^7^ and North Korea.^8^

 As emphasized in the literature, the primary objectives of screening and treating CVD risk factors are to control modifiable risk factors and prevent their early and late complications substantially.^9^ Although the PEN guideline targets populations over 30 years old, health authorities initially prioritized 60-65-year-old inhabitants during the PEN’s implementation in Iran. This demographic is more vulnerable to CVD complications and more resistant to changing unhealthy behaviors. Shahreza, located in the southwest of Isfahan province, where participants had moderate socio-economic status, demonstrated significant improvements in high blood pressure, hyperglycemia, and obesity through health promotion measures and active follow-up after implementing Ira-PEN. These findings align with previous studies showing decreased incidence rates of myocardial infarctions, strokes, and mortality rates due to strokes following Ira-PEN.^10^

 According to DM diagnostic criteria, the prevalence of hyperglycemia in this study exceeded the prevalence reported in the STEPS 2016 study.^11^ However, the prevalence of citizens with FBS ≥ 126 mg/dL significantly decreased from 25.6% to 19.7%. Given that serum glucose control based on HbA1C measurement is more accurate, the prevalence of good glycemic control (HbA1C ≤ 6.5%) increased from 30% to 39.13%, while the STEPS 2021 study in Iran reported a prevalence of HbA1C < 7% at 28.0%.^12^

 Furthermore, the relative frequency of hypertriglyceridemia and hypercholesterolemia reached 42.6% and 14.2% in 2021, respectively. In contrast, the STEPS 2021 study in Iran reported the prevalence of these conditions in the population over 25 years old as 39.7% and 21.2%, respectively. These findings indicate an effective approach for DM and dyslipidemia control in the 60-65-year-old population in the pilot region following the PEN implementation.^13^

 These results represent a significant success, especially considering the challenges posed by the COVID-19 pandemic. COVID-19 infection has been shown to trigger new-onset DM, HTN, and dyslipidemia, with hospitalized patients experiencing disturbances in lipid profiles, including increased triglycerides, LDL-C, and total cholesterol levels.^14^ A rapid assessment by WHO during the pandemic found that PEN training and implementation in primary health systems were disrupted in 65% of low-income countries. The pandemic led to increased behavioral risk factors such as unhealthy diet, alcohol consumption, physical inactivity, and stress, alongside disrupted essential services for DM and HTN control, worsening outcomes.^15^

 In this study, SBP increased significantly in females but decreased in males over five years. DBP showed a reduction trend in both genders, with a statistically significant decrease in males. This finding is consistent with the Tehran Lipid and Glucose Study ^16^ and the Isfahan Healthy Heart Program^17^, which reported higher SBP and HTN rates in females aged over 19 years. The willingness and compliance of women to undergo periodic medical examinations contributed to the increase in female participants after implementing Ira-PEN. Despite the increase in female participation in 2021 compared to 2016, the relative frequency of DM and DBP decreased.

 This study was based on an E-integrated health information system and data bank from the initial screening and PEN training in 2016, which included 22.96% of the total 6,896 inhabitants aged 60-65 years. This may raise concerns about selection bias.

 Unfortunately, data on smoking, alcohol consumption, physical activity, and dietary conditions were not adequately collected at the project’s initiation. Although data were gathered through census sampling, future studies should include a larger population, particularly different age groups, with a comprehensive assessment of other CVD risk factors such as smoking, alcohol consumption, physical inactivity, and unhealthy diet. Additionally, the impact of the COVID-19 pandemic on individual follow-up and treatment adherence should be considered to clarify the efficiency of Ira-PEN interventions.

 Despite the PEN’s recommendation by WHO for controlling NCDs in developing countries, only a few countries have reported the results of PEN implementation, such as Bhutan^7^ and North Korea.^8^ These reports described limited PEN achievements after a short follow-up period (3 months). In contrast, this study explains the achievements of PEN in Iran after a five-year follow-up, particularly during the COVID-19 pandemic when CVD risk factor control deteriorated. This paper aligns with previous studies on PEN interventions in Shahreza, showing controlled rates of cardiovascular events and mortality compared to a control district.^10^

## Conclusion

 In comparing the relative frequency of citizens with CVD risk factors in 2016 and 2021, there was a significant decrease in the relative frequency of citizens with high systolic and diastolic blood pressures and obesity. Additionally, the mean DBP and mean serum levels of FBS decreased significantly. However, the relative frequency of inhabitants with hypercholesterolemia and the mean serum level of total cholesterol increased.

## Acknowledgments

 We are thankful to the administrators, experts, and other respectful health staff in the health network of Shahreza district, particularly Dr. Hojatallah Tanhaei, and the administrators and experts in the Non-Communicable Diseases’ Office of Health Vice-Chancellor at Isfahan University of Medical Sciences, particularly Dr. Mahshid Ahmadian. We are also grateful to the Deputy of Research and Technology of Isfahan University of Medical Sciences for their support.

## Competing Interests

 All authors certify that they have no affiliation or involvement with any organization or entity with any financial or non-financial interest in the topic or material discussed in this article and have no competing interests, financial or non-financial, related to the publication of this article.

## Ethical Approval

 This research was conducted based on the permission of the Research Ethics Committee of Isfahan University of Medical Sciences with code IR.MUI.REC.1399.917. This committee confirms that this research was conducted in accordance with the ethical standards set forth in the 1964 Declaration of Helsinki and its subsequent amendments. This research did not involve any intervention on humans or animals, and ethical issues related to working with animals and humans did not require informed consent, as the entire research process was based on existing data and figures.
